# Short-term outcome of adrenal radiofrequency ablation of adrenal cysts: a single-center experience

**DOI:** 10.1038/s41598-023-30330-7

**Published:** 2023-02-25

**Authors:** Shin Jeong Pak, Yu-mi Lee, Pyo Nyun Kim, Byung-Chang Kim, Jae Won Cho, Won Woong Kim, Tae-Yon Sung, Ki-wook Chung, Suck Joon Hong

**Affiliations:** 1 Department of Surgery, Asan Medical Center, University of Ulsan College of Medicine, Seoul, 05505 Korea; 2 Director of abdominal radiology, Korean Teleradiology Reading Center, Seoul, 06125 Korea; 3 Department of Surgery, Uijeongbu Eulji Medical Center, Eulji University, Uijeongbu-si, 11759 Korea

**Keywords:** Adrenal gland diseases, Adrenal gland diseases

## Abstract

Percutaneous thermal ablation is a minimally invasive treatment for liver, kidney, lung, bone, and thyroid tumors. This treatment also has been used to treat adrenal tumors in patients, but there is no evidence for the efficacy of thermal ablation of adrenal cysts. The present study was performed to analyze the experience of a single center with percutaneous radiofrequency ablation (RFA) of adrenal cysts and to evaluate its efficacy. The present study enrolled all patients who underwent percutaneous RFA for unilateral adrenal cysts from 2019 to 2021. All patients underwent USG-guided percutaneous aspiration of cystic fluid, followed by RFA. A total nine patients with adrenal cysts were included in this study. All of them underwent technically successful percutaneous RFA, with no immediate complication. Follow-up CT 3 months after RFA showed that six of the nine adrenal cysts showed good responses, with reductions in cyst volume ranging from 86.4 to 97.9%. One patient had poor response in the cyst size (volume reduction rate 11.2%). She underwent secondary RFA with resulting that the cyst volume reduced by 91.1%. After a median follow-up period of 17.2 months, eight patients showed no evidence of regrowth. The patient, who showed evidence of regrowth, declined any other treatment and has been under regular surveillance. None of the nine patients developed adrenal insufficiency during the follow-up period. In conclusion, percutaneous RFA is a safe and effective minimally invasive treatment for adrenal cysts, suggesting that percutaneous RFA may be a good alternative option in selected patients.

## Introduction

Adrenal cysts are rare lesions, with an prevalence on autopsy ranging from 0.06 to 0.18%^1–3^. Because adrenal cysts are generally asymptomatic and non-functional, most of them are diagnosed incidentally^2,4,5^. Recent improvements in imaging modalities and their increased use in patient evaluation have increased the incidence rate of incidentally detected adrenal cysts^4,6^. Although adrenal cysts occur at all ages, they are most common during the fourth and fifth decades of life and are threefold more frequent in women than in men^7^. Small, asymptomatic, and hormonally inactive adrenal cysts are usually monitored by periodic follow-up imaging, whereas cysts that are functional or have malignant potential require treatment such as surgery or other interventions^8^.

Huge adrenal cysts are cysts of the adrenal gland > 10 cm in diameter^9^. Huge adrenal cysts may cause abdominal discomfort, gastrointestinal disturbances like nausea and vomiting, or flank pain, and may present as palpable masses due to their size and displacement of adjacent viscera^2^. These cysts may also be a consequence of intracystic bleeding or infection. Less frequent presentations include hypertension or spontaneous rupture of the cyst^5^. Although the treatment of huge asymptomatic adrenal cysts remains unclear, there has been a trend toward performing surgery or other interventions.

Because benign cysts are lined by epithelial cells on the cyst wall and these cells secrete fluid into the cyst cavity, the probability of fluid re-accumulation following cyst evacuation by aspiration alone is high^10–13^. Adrenalectomy is therefore recommended in these patients. Laparoscopic adrenalectomy has several advantages compared with open techniques, such as a shorter hospital stay, less pain, less blood loss, and greater patient satisfaction with cosmesis from small incisions^14^. Nevertheless, laparoscopic adrenalectomy carries a risk of complications and has been associated with mortality in high-risk patients.

Percutaneous thermal ablation is a minimally invasive treatment for liver, kidney, lung, bone, and thyroid tumors^15–19^. This technique has been shown effective and safe for solid organ tumors, including solid tumors of the adrenal glands. In several studies, RFA of functional adrenal tumors resulted in successful biochemical resolution, and RFA of adrenal metastases resulted in local control of these lesions^20–27^. Although percutaneous thermal ablation has shown good outcomes in the treatment of renal and hepatic cysts^28–30^, there is no evidence for thermal ablation of adrenal cyst. The purpose of this study was to demonstrate the experience of our center with percutaneous radiofrequency ablation (RFA) of adrenal cysts and to evaluate its efficacy.

## Methods

### Patient selection and data collection

**Figure 1:**
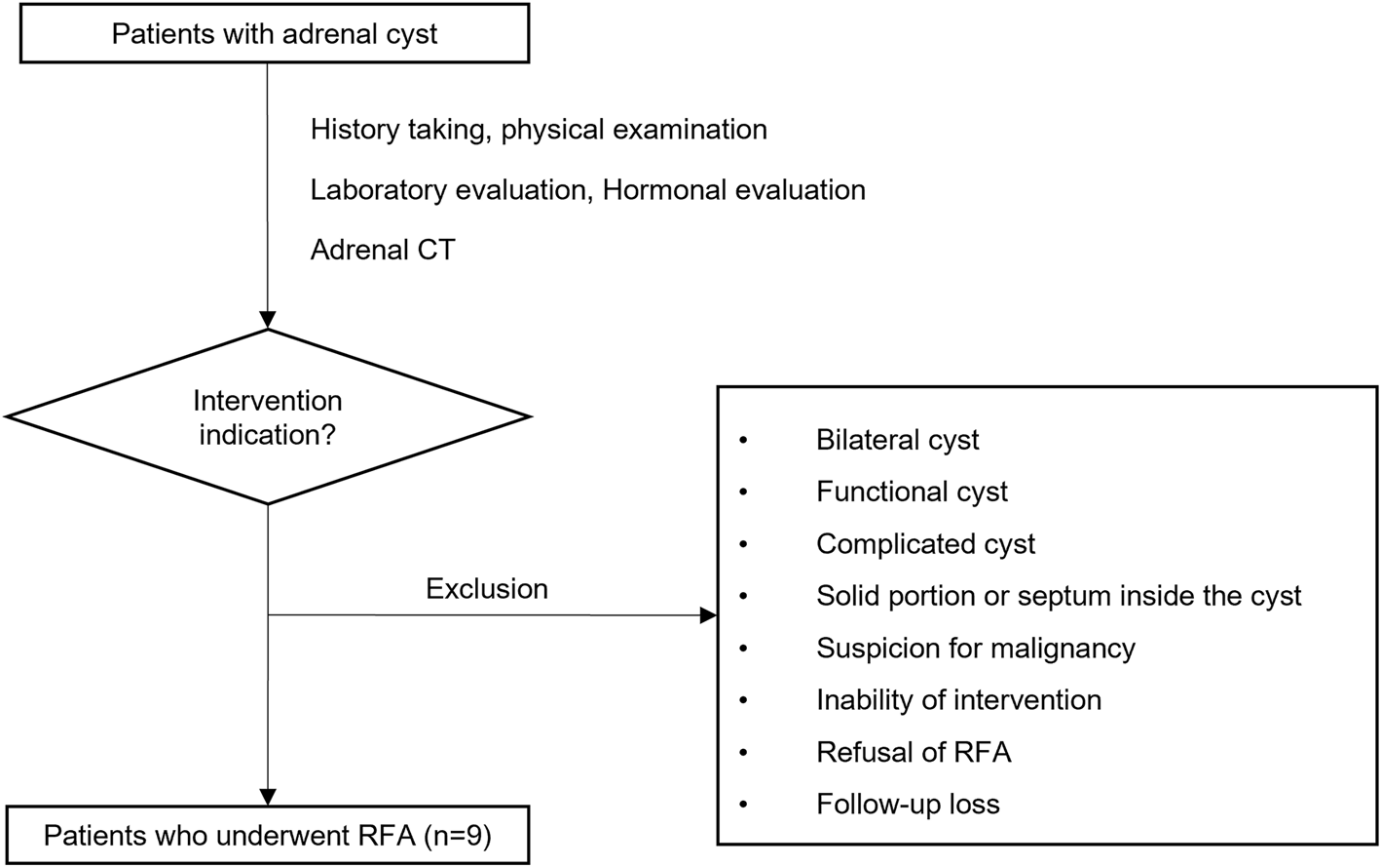
Flow chart of patient selection. RFA, Radiofrequency ablation; Adrenal CT, Adrenal computed tomography.

Patients who underwent percutaneous RFA of unilateral adrenal cysts at Asan Medical Center in Seoul, Korea, from January 2019 to December 2021 were retrospectively identified. Indications of RFA for adrenal cyst were the presence of symptoms at the time of diagnosis, a huge cyst > 10 cm in diameter, or increasing size during follow-up evaluation. Patients with adrenal cysts were excluded (1) if the adrenal cyst was bilateral, functional or complicated, (2) if the cyst had a solid component, (3) if more than two septae were observed on computed tomography (CT), (4) if the cyst was suspected of being malignant, (5) if the RFA procedure was impossible because the adrenal cyst was very close to the surrounding organs (pancreas, liver, spleen or colon), (6) if coagulopathy was present (platelet count < 50 X 10^3^/µL or International Normalized Ratio > 1.5), (7) if the patient declined RFA for an adrenal cyst, or (8) if the patient was lost to follow-up (Fig. 1).

Patient information was collected from their electronic medical records and charts, including their demographic and disease characteristics, procedure details, and outcomes. Demographic characteristics included patient sex, ages at diagnosis and at the procedure, body mass index (BMI), duration of disease from the time of diagnosis, and comorbidities. Disease characteristics included cyst location, functional status of the adrenal disorder, cyst diameter, cyst volume, and symptoms of the disease. Details of the procedure, obtained from procedure reports, included characteristics of the aspirated cyst, aspirated volume, power of the generator, duration of ablation, number of ablation sessions, and temperature of the electrode. Procedure outcomes included VRR on follow-up CT, immediate complications, adrenal insufficiency, readmission, regrowth, re-procedure, length of hospital stay, pain scale, and use of analgesics.

### Percutaneous radiofrequency ablation (RFA)

All procedures were performed under intravenous conscious sedation using 50 μg of Fentanyl citrate (Fentanyl®, BC World Pharm co., Ltd, Seoul, Korea) and local anesthesia. During RFA, the remaining 50 μg of Fentanyl citrate was intravenously injected if a patient complained of abdominal pain.

The insertion point on the skin was marked when a safe needle tract was secured on ultrasound. Approximately 10 mL of 2% lidocaine (Jeil Pharmaceutical; Daegu; Korea) was injected as local anesthesia, from the adrenal capsule to the insertion point along the needle tract. A single, cooled electrode (Cool-tip™ radiofrequency ablation system; Valleylab; Burlington, VT, USA) was inserted into the center of a cyst, followed by the insertion of a 21-gauge Chiba needle (COOK; Bloomington; IN, USA). The cystic content was aspirated through a Chiba needle as much as possible, in order to reduce the ablation time. 10 ml of 2% lidocaine was then injected into the cyst through the Chiba needle five minutes prior to RFA for the purpose of inner epithelial anesthesia.

The ‘hydro-dissection technique’ has been used to avoid thermal injury to adjacent surrounding organs. After the aspiration of the cyst, 5% dextrose in water solution is injected into the tissue plane between the target cyst and the adjacent critical surrounding organs to be protected. The RFA procedure was impossible if the sufficient safety margins of adrenal cysts were not achieved by ‘hydro-dissection technique’.

RFA was performed under ultrasonographic guidance using a 200-W generator (Mygen M-3004; RF Medical; Seoul, Korea) and a single, cooled electrode with a 3-cm active tip by a radiologist (P.N.K.) with 20 years of clinical experience in the field of RFA. For the ablation, the current was maximally elevated from the beginning. Because a large cyst changed in shape after aspiration and presented clover-leaf like configuration, we repeatedly inserted the electrode at unablated portion of a cyst after roll-off of generator power for complete ablation over the entire cyst.

When ablation was ceased, the electrode was removed, and the remaining cystic fluid was aspirated. For monitoring, an iU22 ultrasound system (Philips Healthcare, Bothell, WA, USA) with a 2–5-MHz convex transducer was used.

### Post-treatment assessment

Although ultrasonography (US) was obtained in all patients both before and after RFA, we used CT scans for measuring the cyst volumes. All patients underwent contrast-enhanced, abdominal CT both before and after RFA, and using the usually available scanners (LightSpeed Plus, LightSpeed Ultra16; GE Healthcare, Milwaukee, WI, USA, and Somatom Sensation 16; Siemens Medical Solutions, Forchheim, Germany) with a 5-mm slice thickness.

The two radiologists reached a consensus by assessing the images. The cyst length and width were determined from the axial image showing the largest cyst, and the height was calculated as the total slice numbers showing a cyst. The cyst volume was calculated using the following formula: length x width x height × 4 × 3.14 / 24 = L x W x H / 0.52. After aspiration of the cystic content, we recorded the aspirated volume and the configuration of each cyst (single, septated), and measured its longest and short diameters on USG in order to calculate the volume of the residual fluid. The residual volume was calculated using the following formula: (long diameter + short diameter)3 × 4 × 3.14 / 24.

Complications occurring immediately after RFA were evaluated on contrast-enhanced abdominal-pelvic CT. Complications included inadvertent injury during the RFA procedure, bleeding, infection requiring antibiotics or drainage, and readmission. Complications were classified as major or minor, according to the guidelines of the Standards of Practice Committee of the Society of Interventional Radiology^31^.

**Figure 2:**
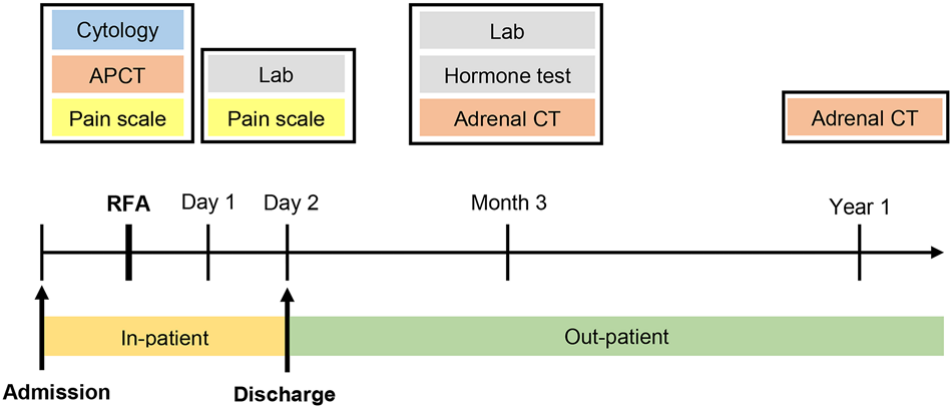
Data collected during this study. RFA, Radiofrequency ablation; APCT, Abdomino-pelvic computed tomography; Lab, Laboratory evaluation; Adrenal CT, Adrenal computed tomography.

The primary outcome was response to treatment, defined as a reduction in volume of the adrenal cyst on radiologic imaging. Responses to treatment on 3 months follow-up adrenal CT scans were defined as good (VRR ≥ 80%), intermediate (40% ≤ VRR < 80%), or poor (VRR ≤ 40%) (Table 1). Patients were followed up 3 months after RFA and every year thereafter by adrenal CT scans (Fig. 2). Cyst regrowth was defined as a cyst volume higher than the volume on the 3 months follow-up CT scan.

**Table 1:** Definitions of responses to treatment.

Response	Description
Good	Adrenal cyst volume on radiologic imaging ≥ 80% lower on 3 months follow-up CT than at baseline
Intermediate	Adrenal cyst volume on radiologic imaging 40% to 80% lower on 3 months follow-up CT than at baseline
Poor	Adrenal cyst volume on radiologic imaging ≤ 40% lower on 3 months follow-up CT than at baseline

### Ethics approval and consent to participate

The institutional review board and ethics committee of Asan Medical Center reviewed and approved the study as per the Helsinki Declaration (institutional review board approval No. 2022-1288). All methods were carried out in accordance with relevant guidelines and regulations. All experimental protocols were approved by the institutional review board and ethics committee of Asan Medical Center. Informed consent was waived by the institutional review board and ethics committee of Asan Medical Center.

## Results

The present study included nine patients with adrenal cysts who underwent RFA from 2019 to 2021 (Table 2). Their mean age was 45.6 years (range 18–79 years). Before RFA, the mean diameter of the adrenal tumors was 10.0 cm (range 6.0–21.0 cm), and the mean initial cyst volume was 278.4 mL (range 122–2717 mL). Of these nine patients, four had adrenal cysts > 10 cm in diameter, two had symptoms such as abdominal discomfort and back pain, one had a recurrent adrenal cyst after percutaneous aspiration, and six had larger adrenal cysts than at initial diagnosis.

**Table 2:** Clinical characteristics and responses to RFA treatment of the nine patients with adrenal cysts.

No	Age (yrs.)	Sex	Size (cm)	Location	Comorbidity	Associated symptoms	Indication for RFA	Aspirated fluid			Procedure time (min)	Analgesics (times)	Pain scale (NRS)	VRR (%)	Treatment response	Regrowth	Re-procedure	Duration of follow-up (months)
								Nature	Volume (cc)	Cytology								
**1**	51	F	10.8	Right	Hypertension	None	Recurrence after aspiration	Serous	300	Negative for malignancy	56	0	0	11.2	Poor	No	Yes (1 times)	22
**2**	79	M	6.2	Right	Hypertension	None	Increased size	Serous	105	Negative for malignancy	65	1	0	95.9	Good	No	No	19
**3**	33	F	11.6	Left	None	None	Huge cyst	Serous	300	-	69	3	5	97.9	Good	No	No	18
**4**	18	F	6.1	Left	None	Abdominal pain	Increased size Symptoms	Serosanguinous	70	Negative for malignancy	60	1	5	97.6	Good	No	No	16
**5**	61	M	6.8	Right	Hypertension	None	Increased size	Serosanguinous	110	-	43	0	0	94.9	Good	No	No	16
**6**	36	F	21.0	Right	None	None	Huge cyst	Serous	2200	Negative for malignancy	72	4	2	89.1	Good	Yes	No	23
**7**	48	F	13.6	Left	None	None	Increased size	Serosanguinous	350	Negative for malignancy	71	3	5	86.4	Good	No	No	15
**8**	36	F	9	Left	None	Back pain Flank pain	Increased size Symptoms	Turbid	300	-	60	1	4	44.5	Intermediate	No	No	10
**9**	48	F	7	Left	Hypertension	None	Increased size	Serous	120	-	77	0	3	71.6	Intermediate	No	No	16

RFA, radiofrequency ablation; VRR, volume reduction rate; NRS, numerical rating scale.

RFA was technically successful in all nine patients, with none experiencing immediate complications, such as inadvertent injury or bleeding. The mean procedure time was 63.7 ± 3.4 min. Following RFA, some patients experienced abdominal pain, which was controlled by medications. The mean pain scale score on the day of RFA was 2.7 ± 0.8 points, decreasing to 0.3 ± 0.2 points 2 days later. Two patients who underwent RFA of right adrenal cysts showed slightly elevated liver enzyme concentrations, but these improved spontaneously without further treatment. The remaining seven patients showed normal laboratory results after RFA (data not shown).

**Figure 3:**
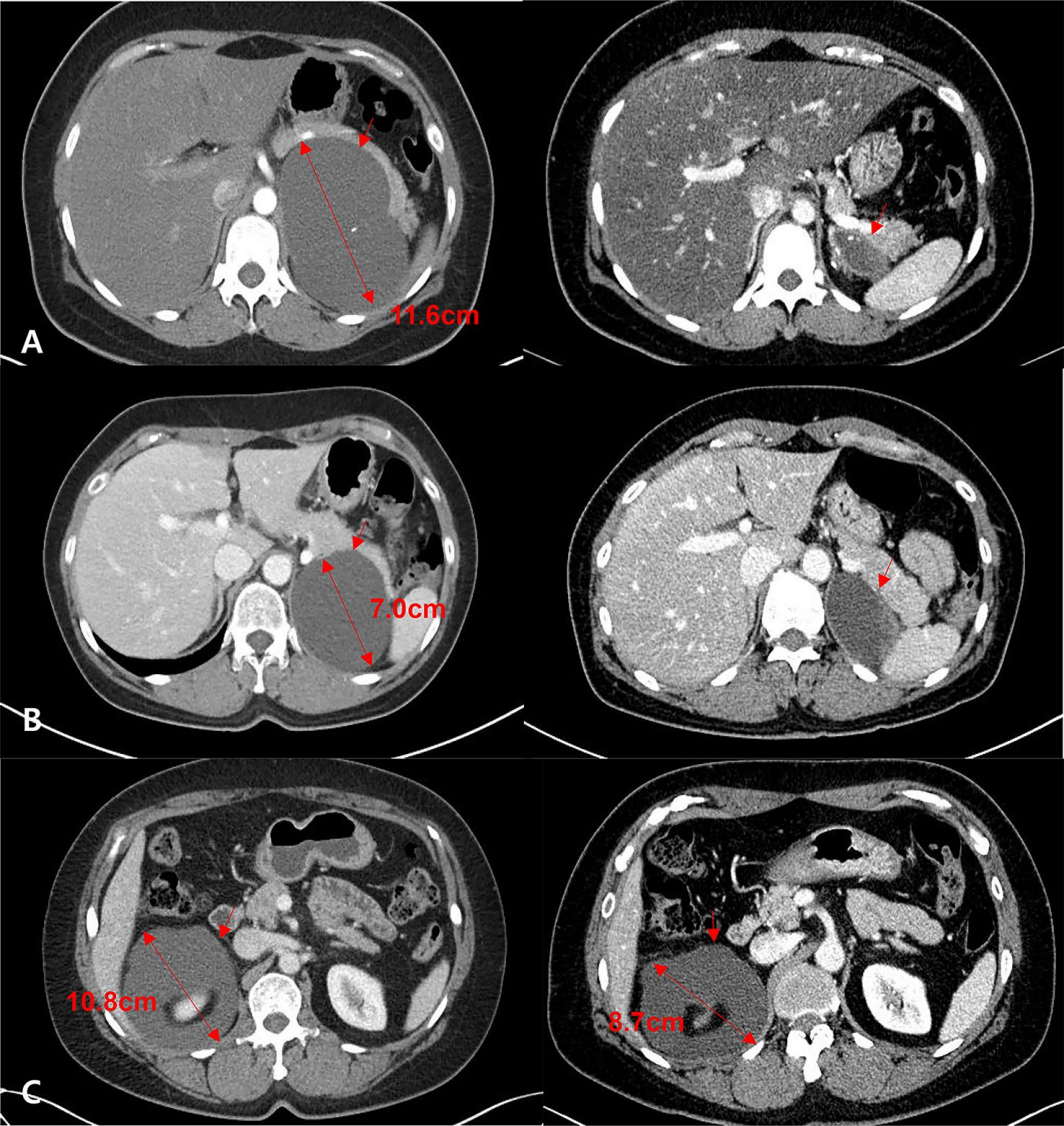
Axial computed tomography (CT) imaging of prior to treatment and 3 months after RFA of (**A**) Patient No. 3, who showed a good response, (**B**) Patient No. 9, who showed an intermediate response, and (**C**) Patient No. 1, who showed a poor response.

Response to treatment was evaluated by comparing initial and 3 months follow-up adrenal CT scans (Table 2). Six of the nine adrenal cysts showed good responses, with reductions in cyst volume ranging from 86.4 to 97.9%, whereas two showed intermediate responses and one had a poor response (VRR 11.2%) (Fig. 3). The latter patient (patient No. 1) was treated with secondary RFA and showed a good response (VRR 91.1%). At final follow-up, the cyst had almost completely disappeared. No patient developed adrenal insufficiency, as determined by adrenal hormone tests 3 months after RFA.

**Figure 4:**
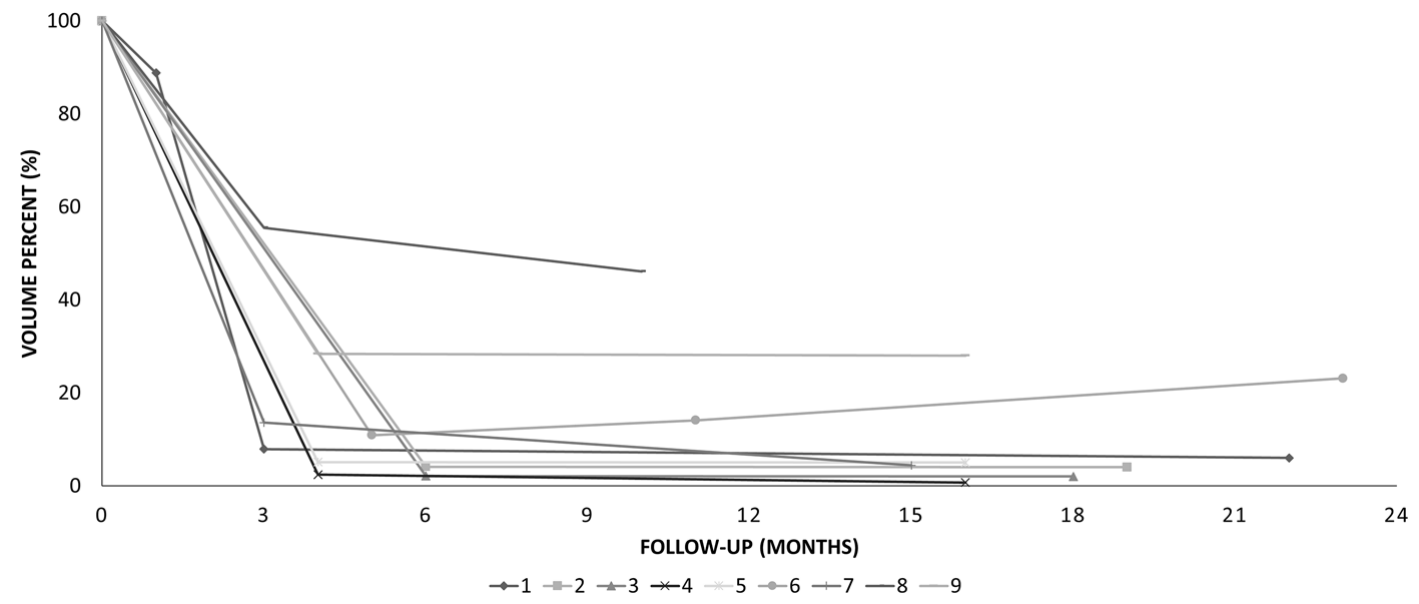
The volume change of cysts over time.

**Figure 5:**
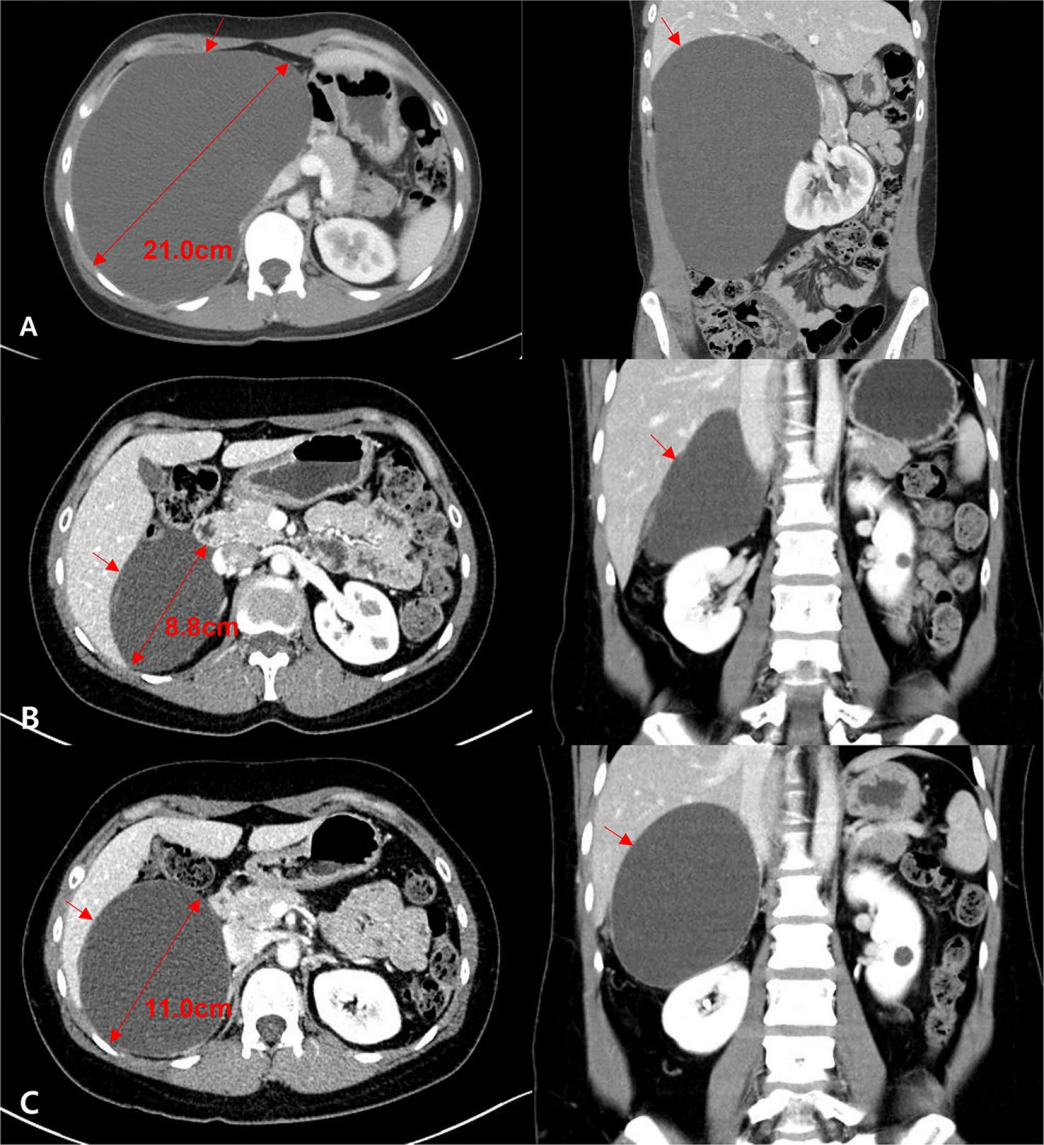
Axial and sagittal computed tomography (CT) imaging of Patient No. 6 (**A**) prior to treatment, (**B**) 3 months after RFA treatment, and (**C**) 12 months after RFA treatment, at the time of cyst recurrence.

The median follow-up period was 17.2 months (range 10–23 months). The mean of volume reduction at 3 and 12 months after RFA was 85% and 87%, except one patient who was treated with secondary RFA (Fig. 4). One patient (patient No.6) experienced a regrowth during the follow-up period (Fig. 5). Although she showed a marked reduction in cyst volume from 21.0 cm before RFA to 8.8 cm at 3 months after treatment (VRR 89.1%), cyst regrowth was observed on the 12-month follow-up CT. This patient declined any other treatment and has been under regular surveillance.

## Discussion

Our case series is the first to show that RFA of adrenal cysts is both a safe and effective. RFA of adrenal cysts was safe in all nine patients, with none experiencing significant complications. CT evaluation at 3 months showed a good response in six patients. One patient, however, experienced cyst regrowth, as shown by an increase in volume during follow-up. None of the nine patients developed adrenal insufficiency.

Minimally invasive treatment of adrenal cysts has been considered for patients who are unable to undergo adrenalectomy. Although the number of published studies is small due to the rarity of this condition, several studies have reported the results of minimally invasive treatments for adrenal cyst. For example, percutaneous aspiration of four of six adrenal cysts showed partial re-accumulation of cyst fluid in two patients^11^. A huge adrenal cyst that recurred after laparoscopic decortication was successfully treated by percutaneous aspiration and ethanol ablation^8^. Although treatment with sclerosing agents has been reported to reduce recurrence rates^10–13^, recurrence within a few months after sclerosis is also common^13^, leading to re-treatment or additional surgery^32^. In addition, the complications associated with the injection of ethanol may lead to various degrees of intoxication and hypotension^33^.

RFA provides homogeneous conduction of heat around the inner wall of a cystic lesion and can cause continuous thermal damage as temperatures are gradually increased^34^. Therefore, this treatment can result in a lower recurrence rate than percutaneous aspiration alone or with sclerosing agents. RFA may also be a good option if a cyst recurs after aspiration. RFA has also been shown to be safe and effective in the treatment of benign cysts, such as hepatic and renal cyst^28–30^.

To our knowledge, no previous studies have assessed the safety and efficacy of RFA in the treatment of adrenal cysts. However, several studies have reported the results of RFA for adrenal solid tumors^21–27^. RFA of functional adrenal tumors resulted in successful biochemical resolution, and RFA of adrenal metastases resulted in local control of these lesions. In addition, none of these patients experienced serious complications, such as injury to adjacent organs or major bleeding. Adrenal insufficiency was reported as a rare complication following RFA, but RFA was superior to surgical adrenalectomy in avoiding this complication. RFA of adrenal cysts was successfully and safely performed in all nine patients in the present study, with all cysts showing a marked reduction in size on immediate post-procedure CT scans, and none of these patients having significant complications. On 3 months follow-up CT scans, six patients showed a good response, defined as a ≥ 80% reduction in adrenal cyst size.

Only one of the nine patients in this study experienced cyst regrowth during the follow-up period. Because of the small number of patients and events, it was difficult to determine the factors associated with this regrowth. During follow-up, however, the reduced cyst sizes were maintained in most of these patients. Only one patient showed a poor response to the first RFA treatment, but this patient showed a good response to a second RFA, with almost complete disappearance of that lesion, which has been maintained through the last follow-up CT. Even in the patient who experienced cyst regrowth and did not undergo additional treatment, the recurred cyst was smaller than the original cyst. Taken together, these findings showed that RFA was highly effective in the treatment of adrenal cysts and has shown excellent results.

The present study has some limitations. First, this study was a single-center retrospective study, which may have resulted in selection bias. Second, the number of patients included in this study was small and the follow-up period was relatively short. This may have overestimated the effectiveness of RFA for adrenal cysts, as it was unclear whether the reduced sizes of adrenal lesions could be maintained over a long period of time. Studies of larger numbers of patients followed-up for a longer period are therefore needed. Finally, this study could not completely rule out the malignant potential of these adrenal cysts. RFA was not performed on patients with suspicious findings on imaging modalities, such as CT or MRI scans. Although the contents of adrenal cysts were aspirated during RFA, with cytology evaluation showing no evidence for malignancy, the possibility that RFA in this study was performed on patients requiring surgery cannot be completely excluded. Proper patient selection is essential for more accurate application of minimally invasive interventions for adrenal cysts.

In conclusion, percutaneous RFA is a safe and effective minimally invasive treatment for adrenal cysts. Percutaneous RFA may therefore be a good alternative option in selected patients.

## Data Availability

The datasets used and/or analyzed during the current study available from the corresponding author on reasonable request.
